# Identification of surrogate endpoints in patients with locoregionally advanced nasopharyngeal carcinoma receiving neoadjuvant chemotherapy plus concurrent chemoradiotherapy versus concurrent chemoradiotherapy alone

**DOI:** 10.1186/s12885-015-1816-6

**Published:** 2015-11-24

**Authors:** Yu-Pei Chen, Wen-Na Zhang, Ling-Long Tang, Yan-Ping Mao, Xu Liu, Lei Chen, Guan-Qun Zhou, Hai-Qiang Mai, Jian-Yong Shao, Wei-Hua Jia, Tie-Bang Kang, Mu-Sheng Zeng, Ying Sun, Jun Ma

**Affiliations:** Department of Radiation Oncology, Sun Yat-sen University Cancer Center, State Key Laboratory of Oncology in South China, Collaborative Innovation Center for Cancer Medicine, 651 Dongfeng Road East, Guangzhou, 510060 People’s Republic of China

**Keywords:** Nasopharyngeal carcinoma, Neoadjuvant chemotherapy, Concurrent chemoradiotherapy, Surrogate endpoint, Overall survival

## Abstract

**Background:**

In the era of intensity-modulated radiotherapy (IMRT), the efficacy of additional neoadjuvant chemotherapy (NACT) to concurrent chemoradiotherapy (CCRT) in locoregionally advanced nasopharyngeal carcinoma (NPC) is currently being investigated in ongoing trials. Overall survival (OS) is the gold standard endpoint in NPC trials. We performed this analysis to identify surrogate endpoints for OS, which could shorten follow-up duration and speed up assessment of treatment effects.

**Methods:**

We retrospectively analysed 208 matched-pair patients with locoregionally advanced NPC receiving NACT+CCRT or CCRT. Progression-free survival (PFS), failure-free survival (FFS), distant failure-free survival (D-FFS) and locoregional failure-free survival (LR-FFS) at 2 and 3 years were assessed as surrogates for 5-year OS according to Prentice’s criteria. The strength of the associations were assessed using Spearman’s rank correlation coefficient.

**Results:**

No significant differences were observed between treatment arms for any surrogate endpoint at 2 years, which rejected Prentice’s second criterion. In contrast, 3-year LR-FFS, PFS, FFS and D-FFS were consistent with all four of Prentice’s criteria; the rank correlation coefficient (0.730) between 3-year PFS and 5-year OS was highest.

**Conclusions:**

3-year PFS, FFS and D-FFS could be valid surrogate endpoints for 5-year OS; 3-year PFS may be the most accurate.

## Background

Nasopharyngeal carcinoma (NPC) is an epithelial malignancy commonly observed in southern China, where the incidence ranges from 15 to 50 per 100,000 [1]. Radiotherapy (RT) is the mainstay treatment modality for non-disseminated NPC. Although locoregional control has improved as a result of the advent of intensity-modulated radiation therapy (IMRT), the prognosis for patients with locoregionally advanced NPC still remains poor due to a high incidence of distant metastasis [2, 3]. As NPC is also relatively chemosensitive, numerous studies have been conducted to evaluate the efficacy of combining chemotherapy and RT in locoregionally advanced NPC. Concurrent chemoradiotherapy (CCRT) with or without adjuvant chemotherapy (AC) has been demonstrated to be most efficacious, and is now recommended as a standard treatment for patients with locoregionally advanced NPC [4, 5]. A meta-analysis indicated that additional neoadjuvant chemotherapy (NACT) could effectively enhance overall survival (OS) and reduce the rate of distant metastasis [6]; however, in the IMRT era, the addition of NACT to CCRT may provide better distant control and may be the most promising strategy [7].

To the best of our knowledge, only two phase II trials [8, 9] comparing NACT+CCRT with CCRT and one phase III trial [10] comparing NACT+CCRT with CCRT+AC have been published so far. However, the phase II trial by Hui et al. [8] reported an improvement in OS with the addition of NACT, while the phase II trial by Fountzilas et al. [9] failed to observe any significant improvement in survival. Only the preliminary results of the phase III trial by Lee et al. [10] have been reported, with no significant differences in OS and progression-free survival (PFS) between arms. The long-term results of phase III trials are awaited to confirm the efficacy of NACT+CCRT in NPC; we are currently conducting two phase III trials (NCT01245959, NCT01872962).

In NPC trials, 5-year OS is commonly used to evaluate the long-term benefits of a treatment. However, a large sample size and a long follow-up period are required to detect statistically significant differences in OS; non-cancer deaths can also impact the measurement of OS. Establishment of valid surrogate endpoints for OS could shorten the duration of a trial, and thus help to assess the results earlier and accelerate the discovery of more effective therapeutic regimens. A set of statistical requirements, known as Prentice’s four criteria, must be met to validate surrogate endpoints [11]. In trials of NPC, the potential surrogate endpoints for OS include PFS, failure-free survival (FFS), distant failure-free survival (D-FFS) and locoregional failure-free survival (LR-FFS). No study has yet assessed whether these endpoints measured at an early time-point (e.g., 2 or 3 years) are useful surrogates for 5-year OS in patients with locoregionally advanced NPC receiving NACT+CCRT versus CCRT alone. Therefore, we performed this analysis to evaluate whether 2- and 3-year PFS, FFS, D-FFS or LR-FFS could be used as surrogate endpoints according to Prentice’s criteria. The cisplatin-fluorouracil (PF) regimen was adopted for additional NACT in the patients in this analysis, as this combination is commonly used in the neoadjuvant phase at our centre and was adopted in the phase III by Lee et al. [10]. The most recent staging system (7th Union for International Cancer Control/American Joint Committee On Cancer [UICC/AJCC]) was applied to define locoregionally advanced NPC.

## Methods

### Patient characteristics

Between January 2003 and December 2007, all 749 patients with newly-diagnosed, biopsy-proven, non-metastatic NPC treated using IMRT at Sun Yat-Sen University Cancer Center were retrospectively reviewed. Exclusion criteria were as follows: (1) patients aged < 18 or > 70 years (*n* = 24); (2) patients without locoregionally advanced NPC (stage III–IV according to the 7th UICC/AJCC staging system; *n* = 253); (3) patients treated without CCRT, and patients treated with AC (*n* = 124). An additional 64 patients who did not receive the PF regimen as NACT or whose chemotherapy regimen information was incomplete were excluded. In the remaining 284 patients with locoregionally advanced NPC who were treated with NACT+CCRT or CCRT alone, we performed one-to-one pair matching [12] between the patients receiving each therapeutic regimen based on the randomization pairing principle for matched-pair analysis. Matching was performed according to age, gender, T category, and N category. Finally, a total of 208 matched-pair patients were analyzed in this study. The clinical features of these patients are shown in Table 1.

**Table 1:** Clinical features of the 208 matched-pair patients with nasopharyngeal carcinoma

Characteristic	NACT+CCRT group	CCRT group
	(*n* = 104, %)	(*n* = 104, %)
Age, years		
≤ 50	86 (82.7)	86 (82.7)
> 50	18 (17.3)	18 (17.3)
Gender		
Male	82 (78.8)	82 (78.8)
Female	22 (21.2)	22 (21.2)
T category ^a^		
T1	7 (6.7)	7 (6.7)
T2	5 (4.8)	5 (4.8)
T3	63 (60.6)	63 (60.6)
T4	29 (27.9)	29 (27.9)
N category ^a^		
N0	13 (12.5)	13 (12.5)
N1	61 (58.7)	61 (58.7)
N2	22 (21.2)	22 (21.2)
N3	8 (7.7)	8 (7.7)
Stage ^b^		
III	68 (65.4)	68 (65.4)
IVA–IVB	36 (34.6)	36 (34.6)

*Abbreviations*: *CCRT* concurrent chemoradiotherapy; *NACT* neoadjuvant chemotherapy

^a^According to the American Joint Committee on Cancer, 7th edition

All patients completed a pretreatment evaluation including a complete patient history, physical examination, haematology and biochemistry profiles, magnetic resonance imaging (MRI) of the neck and nasopharynx, chest radiography, and abdominal ultrasonography; positron emission tomography–computed topography (PET–CT) was performed on 53/208 patients (25.5 %). All patients were restaged according to the 7th UICC/AJCC staging system [13]. All medical records and imaging studies were reviewed to minimize heterogeneity in restaging. Two radiologists specializing in head and neck cancer evaluated the scans separately, and disagreements were resolved by consensus. This study was approved by the ethics committee of Sun Yat-Sen University Cancer Center. As this was a retrospective analysis of routine data, we were granted a waiver of written consent, and verbal consent was obtained from the patients.

### Treatment and follow-up

All patients underwent radical radiotherapy and received a planned total dose of 68–76 Gy (2–2.27 Gy per fractions, five fractions per week). The nasopharyngeal and upper neck tumour volumes were treated using IMRT for the entire treatment course. A conventional anterior or anteroposterior opposing cervical technique was used for the lower neck. Further details of the radiotherapy techniques used at our centre have been reported previously [3]. All patients received cisplatin-based concurrent chemotherapy. Concurrent chemotherapy was initiated on the first day of RT with cisplatin either every three weeks or weekly (based on the opinion of the individual oncologists). No significant differences in long-term survival outcomes and acute toxicity were found between concurrent cisplatin administered weekly or every three weeks [14]. The compliance of concurrent cisplatin was good; over 90 % patients received at least five weeks of concurrent cisplatin when administered weekly, or received at least two cycles of of concurrent cisplatin when administered every three weeks. Additionally, 44/208 (21.1 %) patients received fluorouracil in the concurrent phase, and 104 patients received additional neoadjuvant chemotherapy based on the PF regimen. Reasons for deviation from institutional guidelines included refusal by individual patients or age or organ dysfunction suggestive of intolerance to chemotherapy. When possible, salvage treatments such as intracavitary brachytherapy, surgery and chemotherapy were provided in the event of documented relapse or persistent disease.

The median follow-up was 76 months (range, 6–123 months). Follow-up duration was calculated from the first day of therapy to the day of death or last examination. Patients were examined at least every 3 months during the first 2 years, and 6 months thereafter until death. Endoscopy, CT or MRI scans of the head and neck were performed every 3 months during the first year and annually during years 2–5. Patients with residual or recurrent local disease underwent biopsy to confirm malignancy. Additional tests were ordered when indicated to evaluate for local or distant failure.

### Study endpoints

The standard endpoint was 5-year OS, which was calculated from the first day of treatment to death from any cause. The following potential surrogate endpoints measured at 2 and 3 years were estimated: PFS (calculated from first day of treatment to failure or death from any cause), FFS (calculated from first day of treatment to first failure at any site), D-FFS and LR-FFS (calculated from the first day of treatment to the first distant and locoregional failure, respectively). All events taking place after the 2 or 3-year time-points were censored.

### Statistical analysis

The surrogate endpoints were evaluated using Prentice’s four criteria [11]. A surrogate for a true endpoint should validate a test of the null hypothesis that no correlation exists between the treatment and the true response. Prentice’s four criteria are as follows: (1) treatment is a significant prognostic factor for the true endpoint (e.g., 5-year OS); (2) treatment is a significant prognostic factor for the surrogate endpoint (e.g., 2- and 3-year PFS, FFS, D-FFS or LR-FFS ); (3) the surrogate endpoint is a significant prognostic factor for the true endpoint; and (4) the full effect of the treatment on the true endpoint should be explained by the surrogate endpoint. The actuarial rates for the true and surrogate endpoints were estimated using the Kaplan–Meier method; survival curves were compared using the log-rank test [15]. The adjusted Cox proportional hazard models with backward elimination were used to calculate the hazard ratio (HR) between treatments with respect to each outcome [16]. The following parameters were included in the model as covariates: age (>50 vs. ≤ 50 years), gender (male vs. female), T category (T3–4 vs. T1–2), N category (N1–3 vs. N0), treatment arm (NACT+CCRT vs. CCRT) for Prentice’s criterion one, two and four, and surrogate endpoints (with vs. without events ≤ 2/3 years) for Prentice’s criterion three. Additionally, the Spearman’s rank correlation coefficients (ρ) for the distribution of the candidate surrogates and 5-year OS at the individual level were calculated to assess the strength of the associations, using a bivariate survival model [17]; ρ^2^ reflects the amount of variation explained by the surrogate. SPSS 19.0 software (IBM, Armonk, NY, USA) was used for all analysis.

## Results

Among the 208 matched-pair patients, a total of 58 (27.9 %) patients experienced treatment failure and 56 (26.9 %) patients died during the follow-up period. As expected, locoregional control was good with the adoption of IMRT, and only 21 (10.1 %) patients developed locoregional failure. Most failures occurred at distant sites, with 48 (23.1 %) patients developing distant metastasis.

### Evaluation of surrogate endpoints

#### Prentice’s criterion one

**Fig. 1:**
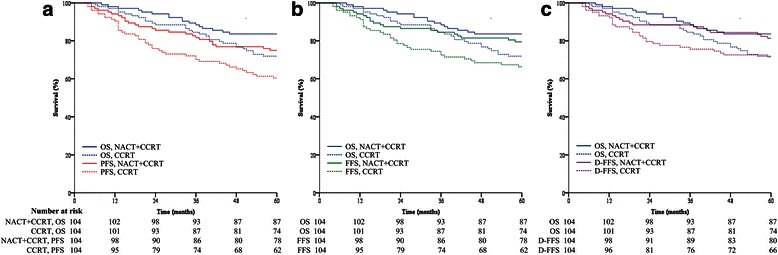
Survival curves for overall survival (OS), and potential surrogate endpoints. Kaplan–Meier survival curves of (**a**) OS and progression–free survival (PFS), (**b**) OS and failure–free survival (FFS), and (**c**) OS and distant failure–free survival (D-FFS) in the neoadjuvant chemotherapy followed by concurrent chemoradiotherapy (NACT+CCRT) group and CCRT alone group. Survival curves were truncated at 5 years. The numbers of patients at risk in each group are provided below the graph

The 5-year OS rate was 72 % in the CCRT group and 84 % in the NACT+CCRT group, with a marginally significant difference (*P*_log-rank_ = 0.050; Fig. 1). The adjusted HR for NACT+CCRT versus CCRT was 0.542 (95 % CI, 0.298–0.987; *P*_Cox_ = 0.045). Therefore, Prentice’s first criterion, that the treatment is a significant prognostic factor for the true endpoint, 5-year OS, was met.

#### Prentice’s criterion two

Though the 2-year PFS and D-FFS rates of the patients in the NACT+CCRT group tended to be better than those of the CCRT group, no statistically significant differences with respect to any surrogate endpoint measured at 2 years were observed between treatments (Table 2). In contrast, compared to patients in the CCRT group, those in the NACT+CCRT group had significantly better 3-year PFS (HR = 0.561, 95 % CI 0.317–0.994; *P*_Cox_ = 0.048), 3-year FFS (HR = 0.527, 95 % CI 0.285–0.975; *P*_Cox_ = 0.041) and 3-year D-FFS (HR = 0.458, 95 % CI 0.230–0.912; *P*_Cox_ = 0.026; Table 2). Treatment was not significantly prognostic for 3-year LR-FFS. Therefore, Prentice’s second criterion that the treatment is a significant prognostic factor for the surrogate endpoint was only met for 3-year PFS, FFS and D-FFS.

**Table 2:** Survival outcomes for the surrogate endpoints at 2 and 3 years

Surrogate endpoint	Events			Survival rates ^a^		
	NACT+CCRT group	CCRT group	Total,	NACT+CCRT group, %	CCRT group, %	*P*-value *
	(*n* = 104, %)	(*n* = 104, %)	No. (%)			
At 2 years						
PFS	15 (14.4)	25 (24.0)	40 (29.2)	87	76	0.078
FFS	14 (13.5)	22 (21.2)	36 (27.3)	87	79	0.134
D-FFS	12 (11.5)	21 (20.2)	33 (15.9)	88	80	0.092
LR-FFS	3 (2.9)	6 (5.8)	9 (4.3)	98	94	0.284
At 3 years						
PFS	19 (18.3)	31 (29.8)	50 (24.0)	83	72	0.049
FFS	16 (15.4)	28 (26.9)	44 (21.2)	85	75	0.040
D-FFS	12 (11.5)	25 (24.0)	37 (17.8)	88	77	0.022
LR-FFS	5 (4.8)	9 (8.7)	14 (6.7)	95	92	0.235

*Abbreviations*: *CCRT* concurrent chemoradiotherapy; *D-FFS* distant failure-free survival; *FFS* failure-free survival; *LR-FFS* locoregional failure-free survival; *NACT* neoadjuvant chemotherapy; *PFS* progression-free survival

^a^The survival rates for the surrogate endpoints were estimated using the Kaplan-Meier method

*P-values were calculated using the log-rank test

#### Prentice’s criteria three and four

Table 3 summarizes the testing of the surrogates for 5-year OS according to Prentice’s criteria three and four. As treatment was not prognostic for all surrogates at 2 years and locoregional failure at 3 years, these endpoints were not included in these tests. When measured at 3 years, PFS, FSS and D-FSS all had a significant impact on 5-year OS (all *P*_Cox_ < 0.001; Table 3).

**Table 3:** Testing of Prentice’s criteria three and four for the potential surrogate endpoints for 5-year OS^a^

Prentice’s criteria	HR (95 % CI)^c^	*P*-value*
Prentice’s criterion three: Is the surrogate endpoint prognostic for 5-year OS?		
Failure or death from any cause ≤ 3 years^b^	39.0 (16.5–92.3)	<0.001
Failure at any site ≤ 3 years^b^	14.9 (7.9–28.1)	<0.001
Distant metastasis ≤ 3 years^b^	18.1 (9.7–33.8)	<0.001
Prentice’s criterion four: Is treatment still prognostic for 5-year OS in patients stratified by the surrogate?		
Patients with failure or death from any cause ≤ 3 years		
Yes	NS	>0.10
No	NS	>0.10
Patients with failure at any site ≤ 3 years		
Yes	NS	>0.10
No	NS	>0.10
Patients with distant metastasis ≤ 3 years		
Yes	NS	>0.10
No	NS	>0.10

*Abbreviations*: *CI* confidence interval; *HR* hazard ratio; *NS* not significant; *OS* overall survival

^a^As treatment was not prognostic for any surrogates measured at 2 years and locoregional failure at 3 years, Prentice’s criterion three and four were not tested for these endpoints

^b^The reference groups were no events ≤ 3 years

^c^Hazard ratios were adjusted for the following parameters using a Cox proportional hazards model by backward elimination: age (>50 vs. ≤ 50 years), gender (male vs. female), T category (T3–4 vs. T1–2), N category (N1–3 vs. N0), surrogates (with vs. without events ≤ 3 years) for Prentice’s criterion three only, and treatment arm (neoadjuvant chemotherapy + concurrent chemoradiotherapy vs. concurrent chemoradiotherapy) for Prentice’s criterion four only

*P-values were calculated using the adjusted Cox proportional hazards model

To assess Prentice’s criterion four, first we tested the hypothesis that 5-year OS was independent of treatment if patients had failure or death due to any cause, failure at any site, or distant metastasis ≤ 3 years. Then we tested the second hypothesis that 5-year OS was independent of treatment if none of the abovementioned events occurred ≤ 3 years. The treatment effect of NACT+CCRT versus CCRT on 5-year OS was not statistically significant for either hypothesis (all *P*_Cox_ > 0.10; Table 3). These findings suggest that the full effect of treatment on 5-year OS may be explained by the potential surrogate endpoints, independent of treatment, and so Prentice’s fourth criterion was also satisfied.

Therefore Prentice’s third criterion, that the surrogate endpoint is significantly prognostic for the true endpoint, and Prentice’s fourth criterion, that the full effect of the treatment on 5-year OS could be explained by the surrogate endpoint, were both met for 3-year PFS, FFS and D-FFS.

### Strength of the associations between surrogate endpoints and 5-year OS

The strength of the associations between the surrogate endpoints and 5-year OS were assessed using the Spearman’s rank correlation coefficient. The association between 3-year PFS and 5-year OS was relatively strong (ρ^2^ = 0.730), while the rank correlation coefficients were 0.661 for 3-year D-FFS and 5-year OS and 0.632 for 3-year D-FFS and 5-year OS.

## Discussion

During the last decade, the advanced therapeutic strategies introduced in the IMRT era have been widely adopted and systematic treatments such as the addition of NACT has been an area of intense research. Currently, two phase III trials are being undertaken by our group to confirm the efficacy of NACT+CCRT in locoregionally advanced NPC (NCT01245959, NCT01872962). The preliminary results of NCT01245959 are yet to be reported. Identification of a valid surrogate endpoint for OS could help to assess long-term survival by observing the surrogate endpoint at earlier time-points. In this retrospective study, we applied the surrogacy criteria devised by Prentice to assess which endpoints could represent useful surrogates for 5-year OS in patients with locoregionally advanced NPC receiving additional NACT. 3-year PFS, FFS and D-FFS were consistent all four of Prentice’s criteria and were validated as surrogate endpoints for 5-year OS, while 3-year LR-FFS and all endpoints measured at 2 years were not confirmed as surrogates. Moreover, compared to FFS and D-FFS, PFS measured at 3 years had the strongest association with 5-year OS (ρ = 0.730). Thus, 3-year PFS, FFS and D-FFS could enable the early assessment of treatment effects on long-term survival using preliminary trial results, with 3-year PFS likely to provide the most accurate prediction of 5-year OS.

With the widespread use of IMRT, the prognosis of patients with NPC has greatly improved, with fewer treatment failures at locoregional sites now occurring [18]. Use of surrogate endpoints measured too early may produce fewer events, which would increase the difficultly of reaching statistical significance and result in less precise prediction of the true endpoint. In this analysis, all endpoints measured at 2 years did not comply with Prentice’s second criterion: no significant differences were observed between treatment groups. During the validation process of surrogate endpoints, the level of significance of treatment effect should be taken into account [19]; endpoints with non significant treatment effects could not serve as good surrogates. However, 2-year PFS and D-FFS trended towards significance, which suggests that future trials recruiting larger numbers of patients, despite the costing more, would observe a higher number of events that may enable 2-year PFS and D-FFS and even FFS, to meet this criterion. Extending the follow-up duration to 3 years, PFS, FFS and D-FFS were validated as surrogate endpoints for 5-year OS. However, due to the excellent locoregional control by IMRT, NACT+CCRT over CCRT alone did not lead to superior LR-FFS, and Prentice’s second criteria was not met for LR-FFS. As the definition of PFS included failure at any site as well as death, which overlaps with the definition of OS, the rank correlation coefficient between 3-year PFS and 5-year OS was relatively high. As it was biased by the insignificant improvement in locoregional control provided by NACT, the predictive ability of 3-year FFS was weakened, and FFS and 5-year OS had a smaller correlation coefficient than 3-year D-FFS and 5-year OS. Thus, PFS, FFS and D-FFS measured at 3 years could all serve as surrogate endpoints for long-term OS; 3-year PFS may be the most appropriate and accurate.

Generally, in a trial with the same number of patients and duration of follow-up, the number of events will be higher or equal for surrogate endpoints (especially PFS) than for OS. Therefore, a valid surrogate endpoint could increase the statistical power to observe significant differences between treatment groups, allowing smaller randomized clinical trials to be carried out and shortening the follow-up period. Use of surrogate endpoints may also permit earlier reporting of a trial and enhance interpretation of the preliminary results, thereby accelerating the development of new treatment strategies (e.g., the addition of NACT to CCRT). Furthermore, when assessment of OS may be contaminated by administration of salvage therapies after treatment failures and non-cancer deaths, surrogate endpoints could reduce the risk of abandoning potentially effective new therapeutic regimens.

A limitation of this analysis is that it is a retrospective study of patients from a single centre. The retrospective nature of the analysis may have confounded the results to a certain extent; using data from randomized trials performed at different centres could make the results more persuasive. However, no long-term results of phase III trials comparing NACT+CCRT with CCRT have yet been reported. In order to reduce bias and reflect typical trial conditions in this study, we applied the newest staging system, included patients with long-term follow-up and performed matched-paired analysis. Our analysis may provide a reference for future trials that are yet to be reported. Another limitation of this study is that the results should be interpreted with caution when applying statistical methods such as Prentice’s criteria to establish surrogate endpoints [20]. As the fourth criterion is formulated in terms of an equivalence setting, which is relatively difficult to meet, not rejecting this criterion is not necessarily definite evidence that the criterion holds [21]. However, PFS, FFS and D-FFS measured at 3 years did satisfy all four criteria, confirming their validity as surrogate endpoints for 5-year OS. Third, we should note that the Prentice’s criteria only ensures that the treatment effect of the true endpoint implies the treatment effect also on the surrogates; it does not ensure the converse [22]. It means that a signicant treatment effect on 3-year PFS, FFS or D-FFS can not promise a significant treatment effect on the 5-year OS; uncertainty exists in using surrogate.

It is important to keep in mind that extrapolation of a surrogate endpoint validated for a specific therapeutic regimen to other regimens with totally different mechanisms of action may not be reliable. In this study, NACT based on the PF regimen was investigated; this regimen was also adopted for NACT in a phase III trial that compared NACT+CCRT with CCRT+AC in locoregionally advanced NPC and recently published preliminary results [10]. The two ongoing phase III trials by our group are designed to evaluate the taxane-PF regimen and gemcitabine-cisplatin regimen for NACT, respectively. Though application of the surrogate endpoints identified in this study may be suitable for these traditional chemotherapeutic agents, surrogate endpoints should be directly verified in trials evaluating targeted or immunological therapies, which have totally different pharmacodynamic profiles.

In conclusion, this analysis identified 3-year PFS, FFS and D-FFS as valid surrogate endpoints for 5-year OS in patients with locoregionally advanced NPC receiving NACT+CCRT versus CCRT alone. 3-year PFS may be more useful for early assessment of treatment effects. Long-term follow-up is still required in future trials to confirm the efficacy of the treatments and monitor unexpected late adverse effects. Future trials should continue to be designed with OS the main end-point in conjunction with potential surrogate endpoints.

## Conclusions

We performed a retrospective study to eatimate whether 2- and 3-year PFS, FFS, D-FFS, and LR-FFS could be used as surrogate endpoints for 5-year OS in patients with locoregionally advanced NPC receiving NACT+CCRT versus CCRT alone. We demonstrated that 3-year PFS, FFS, and D-FFS could be valid surrogates, while 3-year PFS may be the most accurate. Establishment of these surrogate endpoints for OS could shorten the duration of a trial, thus help us assess the results earlier, and accelerate the finding of effective therapeutic regimens.

## Abbreviations

NPC: nasopharyngeal carcinoma
RT: radiotherapy
IMRT: intensity modulated radiation therapy
CCRT: concurrent chemoradiotherapy
AC: adjuvant chemotherapy
NACT: neoadjuvant chemotherapy
OS: overall survival
PFS: progression-free survival
FFS: failure-free survival
D-FFS: distant failure-free survival
LR-FFS: locoregional failure-free survival
PF: cisplatin-fluorouracil
UICC/AJCC: International Cancer Control/American Joint Committee On Cancer
SYSUCC: Sun Yat-Sen University Cancer Center
HR: hazard ratio
